# The T_2_ structure of polycrystalline cubic human insulin

**DOI:** 10.1107/S2059798323001328

**Published:** 2023-04-11

**Authors:** Dimitris P. Triandafillidis, Fotini Karavassili, Maria Spiliopoulou, Alexandros Valmas, Maria Athanasiadou, George Nikolaras, Stavroula Fili, Paraskevi Kontou, Matthew W. Bowler, Christos T. Chasapis, Robert B. Von Dreele, Andrew N. Fitch, Irene Margiolaki

**Affiliations:** a Section of Genetics, Cell Biology and Development, Department of Biology, University of Patras, 26504 Patras, Greece; b European Molecular Biology Laboratory, 71 Avenue des Martyrs, 38042 Grenoble, France; c Institute of Chemical Biology, National Hellenic Research Foundation, 11635 Athens, Greece; d Advanced Photon Source, Argonne National Laboratory, 9700 South Cass Avenue, Lemont, IL 60439, USA; e European Synchrotron Radiation Facility, BP 220, 38043 Grenoble CEDEX 9, France; Fonds National de la Recherche, Luxembourg

**Keywords:** human insulin, cubic polymorph, X-ray powder diffraction, synchrotron radiation

## Abstract

The polymorphism of human insulin upon pH variation was characterized *via* X-ray powder diffraction, employing a crystallization protocol previously established for co-crystallization with phenolic derivatives. Two distinct rhombohedral (*R*3) polymorphs and one cubic (*I*2_1_3) polymorph were identified with increasing pH, corresponding to the T_6_, T_3_R_3_^f^ and T_2_ conformations of insulin, respectively. The structure of the cubic T_2_ polymorph was determined *via* multi-profile stereochemically restrained Rietveld refinement at 2.7 Å resolution. This constitutes the first cubic insulin structure to be determined from crystals grown in the presence of zinc ions, although no zinc binding was observed. The differences of the polycrystalline variant from other cubic insulin structures, as well as the nature of the pH-driven phase transitions, are discussed in detail.

## Abstract 2

Characterization of the polymorphism of human insulin upon pH variation and structure determination of the cubic polymorph *via* Rietveld refinement are reported.

## 1. Introduction

The story of the structural characterization of insulin has been tightly coupled with the development of macromolecular crystallography since the late 1920s (Vijayan, 2002 ▸). After a century of crystallographic research, insulin has proven to be one of the most polymorphic proteins, exhibiting a variety of molecular conformations, crystal forms and metal- and ligand-binding abilities.

At its core, insulin is a small protein comprising 51 amino acids in two polypeptide chains: A and B. Depending on the physicochemical environment, the N-terminal region of chain B can be found in a helical (R), intermediate (R^f^) or extended (T) conformation, although only the extended conformation is observed for monomeric insulin in solution. The helical and intermediate conformations, which exhibit higher physicochemical stability (Rahuel-Clermont *et al.*, 1997 ▸), are only observed in hexameric assemblies of insulin coordinated by divalent cations, physiologically zinc. The resulting hexamers can adopt one of the following combinations of conformations: T_6_, 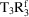 or R_6_ (Kaarsholm *et al.*, 1989 ▸). Although the T_6_ conformation is predominantly observed in solution, the 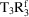 state can be stabilized in the presence of halides, pseudo-halides or organic carboxylates, while the further addition of phenol derivatives can stabilize the R_6_ conformation (Dunn, 2005 ▸; Whittingham *et al.*, 1995 ▸).

Two highly similar crystal forms or polymorphs of rhombo­hedral insulin have been observed to date for the T_6_ (Adams *et al.*, 1969 ▸) and 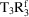 (Bentley *et al.*, 1976 ▸) molecular conformations, respectively, while insulin hexamers complexed with phenolic ligands in the R_6_ conformation have been observed in a variety of crystal polymorphs, as briefly summarized by Spiliopoulou, Valmas *et al.* (2020 ▸). In the absence of zinc cations and in the millimolar concentration range insulin monomers cannot form hexamers and instead crystallize as dimers, predominately with cubic symmetry. This cubic polymorph was first identified by Abel *et al.* (1927 ▸), while the zinc-free nature of these crystals was only recognized after 31 years (Schlichtkrull, 1958 ▸) and the first porcine cubic insulin structure was solved a further 20 years later (Dodson *et al.*, 1978 ▸).

Owing to its high solvent content [∼65%(*v*/*v*)], the cubic polymorph has served as an ideal system to explore pH-dependent conformational changes (Gursky, Badger *et al.*, 1992 ▸), monovalent metal-binding sites (Gursky, Li *et al.*, 1992 ▸) and the protonation states of several structurally crucial amino acids (Diao, 2003 ▸; Ishikawa, Chatake, Morimoto *et al.*, 2008 ▸; Ishikawa, Chatake, Ohnishi *et al.*, 2008 ▸). More recently, the cubic polymorph of the human insulin (HI) variant was reported as a byproduct of co-crystallization efforts with polysialic acid (Timofeev *et al.*, 2010 ▸), while multiple mutants and engineered constructs in a cubic setting are now available in the Protein Data Bank (PDB).

Our interest in the cubic insulin polymorph was a result of our crystallization studies of HI–ligand complexes in the search for stable and densely packed polymorphs that could serve as more potent microcrystalline antidiabetic formulations (Spiliopoulou, Valmas *et al.*, 2020 ▸; Karavassili *et al.*, 2017 ▸). Using X-ray powder diffraction (XRPD), we exploit the observation of novel crystalline polymorphs of insulin as a first strong indication of ligand binding. Thus, in our efforts to ascertain which polymorphs are suggestive of ligand binding, we pursued the crystallization of HI *via* our established protocol but in the absence of any ligands. During these experiments we observed the cubic HI polymorph and, motivated by the impressive quality of the collected XRPD data and the lack of a wild-type human cubic insulin structure in the PDB at the time, we set forth to determine its structure to explore the potential differences between cubic insulins from different species.

## 2. Experimental

### 2.1. Materials

Recombinant human insulin (batch No. SPIN-05-101B) was provided by Novo Nordisk A/S. Zinc acetate (CAS No. 5970-45-6) was purchased from Sigma–Aldrich (now part of Merck KGaA). Sodium thiocyanate (CAS No. 540-72-7) was purchased from Fluka Chemie (now part of Merck KGaA). Disodium hydrogen phosphate (CAS No. 10028-24-7) was purchased from PanReac AppliChem ITW Reagents. Potassium dihydrogen phosphate (CAS No. 7778-77-0) was purchased from Honeywell International Inc. Deionized water from an Aquatron A4D system equipped with a Zalion 200.4 column was used in all experiments.

### 2.2. Crystallization experiments

Human insulin was crystallized employing a previously established protocol for ligand co-crystallization experiments (Fili *et al.*, 2015 ▸; Karavassili *et al.*, 2012 ▸, 2017 ▸; Spiliopoulou, Valmas *et al.*, 2015 ▸, 2021 ▸; Triandafillidis *et al.*, 2020 ▸).

#### 2.2.1. Buffer preparation

Two phosphate solutions were prepared: disodium hydrogen phosphate (Na_2_HPO_4_) and potassium dihydrogen phosphate (KH_2_PO_4_) at 2 *M* concentration. The phosphate-buffer stocks were mixed in appropriate ratios under a pH meter to create a systematic pH gradient of 2 *M* phosphate buffers. A total of 21 buffers were prepared with pH values between 4.5 and 8.5.

#### 2.2.2. Crystallization

For 20 samples, 380 mg lyophilized zinc-free insulin was dissolved in 20 ml double-distilled water (ddH_2_O). 2.31 ml 0.01 *M* zinc acetate solution was added to form insulin hexamers and the solution was aspirated lightly until no clouding was observed. After 5 min, 0.299 ml 1 *M* sodium thiocyanate solution was added to the protein mixture.

**Figure 1:**
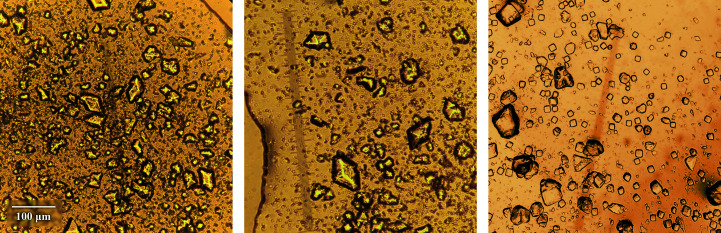
From left to right: polycrystalline HI samples corresponding to polymorphs *R*3_T6_ (nl18, pH 6.31), *R*3_T3R3_ (nl116, pH 7.06) and *I*2_1_3 (nl218, pH 7.70). The scale bar corresponds to all panels.

For each sample, 1 ml of the protein mixture along with 0.25 ml phosphate buffer solution were placed in an Eppendorf tube, resulting in a final protein concentration of 13.50 mg ml^−1^ or 2.32 m*M* (Table 1 ▸). Samples were stored at room temperature and crystals started appearing after 1–5 days (Fig. 1 ▸), depending on the pH. Powder diffraction patterns were collected at least one month after crystallization experiments.

Each sample was labeled with a unique identifier starting with ‘nl’ (*i.e.* no ligand) followed by the experiment series number and an incremental sample number in order of increasing pH. For example, nl212 corresponds to sample 12 of the second crystallization experiment.

A variation of the sample pH was observed with time. Throughout this study, ‘starting pH’ will refer to the pH of the crystallization buffer prior to mixing with the protein mixture, while ‘final pH’ will refer to the pH of the sample prior to diffraction experiments, which was determined by directly measuring the pH of the supernatant crystallization solution.

### 2.3. X-ray powder diffraction (XRPD) measurements

#### 2.3.1. Sample preparation

Prior to XRPD data collection, the polycrystalline protein slurries were transferred to borosilicate glass capillaries of 1 mm diameter and 0.1 mm thickness. Each capillary was first filled with mother liquor, *i.e.* the supernatant crystallization solution, and an amount of polycrystalline slurry was then transferred using a pipette or a syringe. The capillaries were then centrifuged to enhance crystal packing and topped up with more slurry if needed. A sample volume with a height of ∼1 cm is required per capillary, but preferably much more, depending on sample availability. Excess mother liquor was removed with a syringe and the capillaries were cut and sealed with silicone vacuum grease to prevent dehydration. Each sample was mounted onto the goniometer head of the diffractometer and rotated during data collection to ensure better powder averaging.

#### 2.3.2. Data collection

High-resolution XRPD data were collected on the ID22 beamline (Fitch, 2004 ▸; Dejoie *et al.*, 2018 ▸) at the European Synchrotron Radiation Facility (ESRF), Grenoble, France in Debye–Scherrer mode at room temperature employing a nine-channel LaBr_3_ scintillator detector equipped with nine Si(111) crystal analyzers, during two separate experiments [series 1, λ = 1.29974 (1) Å; series 2, λ = 1.299995 (3) Å]. Each capillary was measured at multiple adjacent positions along the capillary axis to minimize radiation-damage effects, collecting two successive scans per position. The detector was translated at 25° min^−1^ and each sample position was exposed to the beam for 2 min per scan. All first and second scans from all capillary positions were then merged separately. Only the merged first scans were used for further analysis since radiation damage was observed in subsequent scans. Each detector channel was also individually inspected for signs of radiation damage, such as peak shifts or loss of diffraction intensity, and was excluded from merging if necessary.

Additional XRPD data were collected on an in-house X’Pert Pro diffractometer (Malvern Panalytical) in Debye–Scherrer geometry with Cu *K*α radiation [λ = 1.540585 (3) Å; Hölzer *et al.*, 1997 ▸] at room temperature employing a PIXcel 1D detector. Each sample was repeatedly measured at a single capillary position for ∼24 scans per sample. The detector was translated at 1° min^−1^ and the sample was exposed for 30 min per scan. The individual scans were inspected for signs of radiation damage and were then averaged to enhance the data statistics. No noticeable radiation damage was observed even after 12 h of data collection, due to the significantly lower beam brilliance.

#### 2.3.3. Data processing

For each crystallization series, the XRPD patterns were grouped manually *via* surface plots or semi-automatically *via* principal component analysis using *HighScore Plus* (Degen *et al.*, 2014 ▸). Representative patterns from each cluster were then indexed using *DICVOL04* (Boultif & Louër, 2004 ▸).

Pawley refinement (Pawley, 1981 ▸) was performed for each data set in *HighScore Plus* to extract peak intensities and accurately characterize the lattice dimensions and peak shape parameters. The peak profile was modeled using a pseudo-Voigt function (Thompson *et al.*, 1987 ▸) with full-width at half-maximum (FWHM) and asymmetry type following the Caglioti–Paoletti–Ricci (Caglioti *et al.*, 1958 ▸) and Finger–Cox–Jephcoat (Finger *et al.*, 1994 ▸) models, respectively.

### 2.4. Structure refinement

Six data sets were selected for structure refinement of the, at the time, unknown human variant of the cubic insulin polymorph.

#### 2.4.1. Data-set selection

Candidate data sets were initially subjected to multi-profile Pawley refinement in *PRODD* (Wright & Forsyth, 2000 ▸; Wright, 2004 ▸). In this process, intensities are extracted *via* a single refinement in which each diffraction profile is calculated as a sum of overlapping reflections, the intensities of which are variables in a least-squares procedure. All data sets are fitted using the same integrated intensities for each pattern but with different unit-cell parameters. This preliminary analysis verifies the isomorphism of the data sets employed for structure refinement by assessing the quality of the fit and the discrepancies in refinement statistics (for example *R*_wp_, χ^2^) across all data sets, while enabling the extraction of a more precise intensity set with higher effective completeness (Basso *et al.*, 2005 ▸; Margiolaki *et al.*, 2007 ▸; Margiolaki & Wright, 2008 ▸; Besnard *et al.*, 2007 ▸; Wright *et al.*, 2007 ▸; Margiolaki, 2019 ▸). A single set of intensities is extracted *via* this process.

Following preliminary analysis, a total of six data sets were selected for structure refinement: five synchrotron data sets and one laboratory data set (Supplementary Table S1). Reflections with a reasonable signal-to-noise ratio [*I*/σ(*I*) > 2] could be observed up to a *d*-spacing resolution of 2.7 Å.

#### 2.4.2. Rietveld refinement

In order to initiate structure refinement, we employed PDB entry 9ins (Gursky, Li *et al.*, 1992 ▸), corresponding to porcine cubic insulin, as a starting model. The model was initially stripped of solvent molecules and alternative conformations. In addition, ThrB30 was substituted with an alanine, which is physiologically found in HI. Owing to the absence of any clear indications of structural heterogeneity upon pH variation, a six-histogram, stereochemically restrained Rietveld refinement (Rietveld, 1969 ▸) was performed using *GSAS* (Larson & Von Dreele, 2004 ▸) to extract an average structural model of the cubic human insulin polymorph (Margiolaki, 2019 ▸; Margiolaki & Wright, 2008 ▸; Spiliopoulou, Triandafillidis *et al.*, 2020 ▸). This procedure has previously been applied to sperm whale metmyoglobin (Von Dreele, 1999 ▸), hen and turkey egg-white lysozyme (Von Dreele, 2001 ▸, 2005 ▸, 2007 ▸; Basso *et al.*, 2005 ▸; Margiolaki *et al.*, 2005 ▸), human and bovine insulin (Von *et al.*, 2000 ▸; Margiolaki *et al.*, 2013 ▸), human ponsin domain SH3.2 (Margiolaki *et al.*, 2007 ▸) and a pharmaceutical peptide, octreotide (Spiliopoulou, Karavassili *et al.*, 2021 ▸; Fili *et al.*, 2019 ▸).

A single set of unit-cell parameters was extracted from the first data set alone, while lattice differences with subsequent data sets were modeled *via* refinable hydroelastic strain terms of the reciprocal metric tensor elements (Larson & Von Dreele, 2004 ▸). The same peak profile used for Pawley refinement was also used for Rietveld refinement. Peak shape parameters, background coefficients, zero-shift, unit-cell and lattice-strain terms were first optimized in a multi-profile Le Bail refinement (Le Bail *et al.*, 1988 ▸) in *GSAS* and then further refined during Rietveld refinement.

The solvent-content contribution to the diffracted intensities was accounted for using the exponential scaling model (Moews & Kretsinger, 1975 ▸; Weichenberger *et al.*, 2015 ▸), which is a direct application of Babinet’s principle to the calculated structure factors. Although more sophisticated masking methods are typically employed in macromolecular crystallo­graphy, this approach has proven to be accurate enough for the medium resolution of macromolecular XRPD data (Von Dreele, 1999 ▸, 2005 ▸; Margiolaki *et al.*, 2005 ▸, 2013 ▸).

In order to reduce the number of refinable parameters and to increase the robustness of the refinement process, protein atoms were described using the flexible rigid-body (FRB) approach (Margiolaki *et al.*, 2013 ▸; Von Dreele, 2019 ▸; Spiliopoulou, Karavassili *et al.*, 2021 ▸; Fili *et al.*, 2019 ▸). Here, each residue is considered as a rigid body with its origin fixed at the location of the C^α^ atom and its orientation described *via* a quaternion, thus requiring a total of only six refinable parameters. The side chain and backbone carbonyl of the residue are considered to be flexible components; thus, additional refinable torsion angles are introduced for these. Bond lengths and angles are kept fixed at typical values. This approach requires ∼66% fewer refinable parameters than a free-atom refinement. In addition, the isotropic temperature factors used for refinement were constrained across all protein and solvent atoms, respectively.

After most non-atomic model parameters (for example histogram scale factors, solvent coefficients, profile parameters *etc.*) reached reasonable values, total OMIT maps (Bhat, 1988 ▸; Bhat & Cohen, 1984 ▸) were generated using *SFCHECK* (Vaguine *et al.*, 1999 ▸) to evaluate the fit of the structure to the electron density. OMIT maps are typically the preferred type of map for macromolecular powder diffraction, due to the inherent model bias in the observed structure factors extracted *via* the Rietveld method (Toby, 2019 ▸; Spiliopoulou, Triandafillidis *et al.*, 2020 ▸). However, due to the low effective completeness of the multi-data-set refinement (Supplementary Fig. S1) resulting from the exactly overlapping peaks of the cubic crystal system, standard 2*F*_o_ − *F*_c_ maps were also employed to aid the model-building process.

Using OMIT and 2*F*_o_ − *F*_c_ maps, water molecules were gradually added to the structure in *Coot* (Emsley & Cowtan, 2004 ▸; Emsley *et al.*, 2010 ▸). The model was periodically subjected to stereochemical checks using *PROCHECK* (Laskowski *et al.*, 1993 ▸) and *MolProbity* (Chen *et al.*, 2010 ▸; Williams *et al.*, 2018 ▸) to highlight incorrectly built regions of the structure. The final model reached a total *R*_wp_ = 6.94% and χ^2^ = 2.06 and was deposited in the PDB under accession code 7qac.

### 2.5. Molecular-dynamics simulations

In order to assess the conformational plasticity of the polycrystalline cubic insulin structure, coarse-grained molecular-dynamics simulations were performed. For comparison, additional simulations were performed on HI structures solved *via* conventional single-crystal techniques: a cubic T_2_ structure (PDB entry 3i40; Timofeev *et al.*, 2010 ▸), a rhombohedral T_6_ structure (PDB entry 1mso; Smith *et al.*, 2003 ▸) and an orthorhombic T_2_ structure (PDB entry 1b9e; Yao *et al.*, 1999 ▸).

Initially, the structures were energy-minimized to avoid steric clashes or invalid stereochemistry using the *mdrun* engine in *GROMACS* (Pronk *et al.*, 2013 ▸). Molecular-dynamics simulations were performed using the *UNRES* web server (Czaplewski *et al.*, 2018 ▸) for coarse-grained protein dynamics at a temperature of 300 K for a total of 100 ns. *UNRES* relies on the highly reduced united-residue model with only two interaction sites: the united peptide backbone, which consists of pseudobond-linked C^α^ atoms, and united side chains, which are pseudobond-linked to the respective C^α^ atoms (Khalili *et al.*, 2005 ▸). The coarse-grained *UNRES* force field has been developed on a solid statistical-mechanical basis by expanding the potential of mean force of polypeptide-containing systems in water into a cluster-cumulant series and by parameterization of the series terms for simple model systems (Sieradzan *et al.*, 2015 ▸). During the simulations, the root-mean-square deviations of the structures with respect to the initial crystal structures began to stabilize after 20 ns.

## 3. Results

### 3.1. Insulin polymorphism as a function of pH

**Figure 2:**
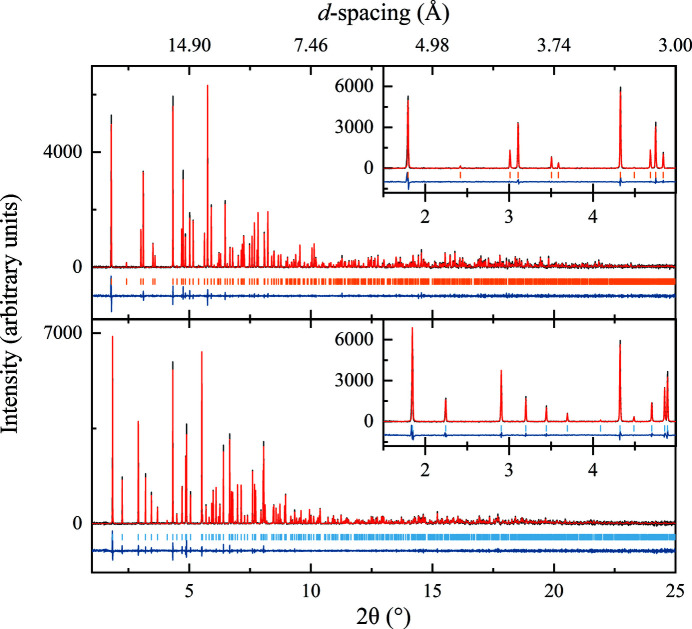
Pawley refinement of polycrystalline human insulin for polymorphs *R*3_T6_ (upper panel; final pH 5.87, *R*_wp_ = 7.61%, χ^2^ = 1.48) and *R*3_T3R3_ (lower panel; final pH 6.84, *R*_wp_ = 6.10%, χ^2^ = 1.40). Black, red and blue lines represent the experimental data, the calculated profile and the difference between them, respectively. The vertical lines correspond to indexed peak positions. The insets correspond to magnifications of the low-resolution region, highlighting the differences in peak positions despite the closely related unit-cell parameters. Data were collected on beamline ID22 at ESRF [λ = 1.29974 (1) Å] at room temperature.

Three polymorphs of HI were observed at different pH values. At acidic pH (final pH 5.33–6.77) HI adopts a rhombohedral symmetry (space group *R*3; Fig. 2 ▸, upper panel) with unit-cell parameters *a* = 82.9870 (5), *c* = 34.0604 (3) Å for sample nl15 (*R*_wp_ = 7.61%, χ^2^ = 1.4844). The unit-cell parameters suggest that HI adopts the extended T_6_ hexameric molecular conformation, based on the literature (Adams *et al.*, 1969 ▸; Blundell *et al.*, 1971 ▸; Smith *et al.*, 2003 ▸). From now on, this polymorph will be referred to as *R*3_T6_.

Around neutral pH (final pH 6.76–7.36) HI again adopts a rhombohedral symmetry (space group *R*3; Fig. 2 ▸, lower panel), but with unit-cell parameters *a* = 80.5817 (6), *c* = 37.6980 (3) Å for sample nl114 (*R*_wp_ = 6.10%, χ^2^ = 1.4038). The unit-cell parameters suggest that insulin adopts the intermediate 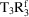 hexameric conformation (Bentley *et al.*, 1976 ▸; Ciszak & Smith, 1994 ▸; Von Dreele *et al.*, 2000 ▸; Frankær *et al.*, 2012 ▸). From now on, this polymorph will be referred to as *R*3_T3R3_.

At basic pH (final pH 7.35–8.43) HI adopts a cubic symmetry (space group *I*2_1_3) with unit-cell parameter *a* = 78.8635 (3) Å for sample nl218 (*R*_wp_ = 15.26%, χ^2^ = 1.6777). This polymorph has previously been observed for porcine (Badger *et al.*, 1991 ▸; Dodson *et al.*, 1978 ▸) and bovine insulin (Gursky, Li *et al.*, 1992 ▸; Gursky, Badger *et al.*, 1992 ▸) and more recently for the human variant (Timofeev *et al.*, 2010 ▸).

**Figure 3:**
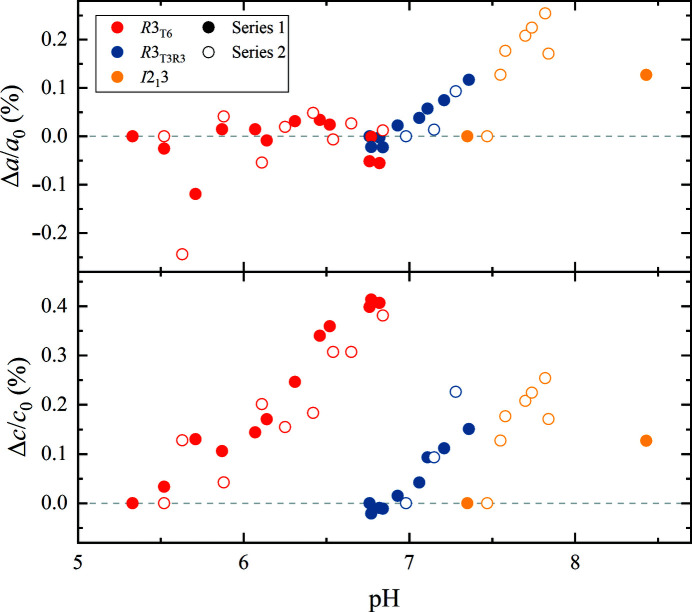
Evolution of unit-cell parameters for the three insulin polymorphs as a function of pH. The percentage change was calculated with respect to the first (*i.e.* lowest pH) sample of each polymorph for each crystallization series, independently. pH values refer to final pH prior to data collection.

For each of the three polymorphs, a systematic variation in unit-cell parameters was observed as a function of pH (Fig. 3 ▸). Polymorph *R*3_T6_ exhibited a 0.41% (∼0.14 Å) increase along the *c* axis over 1.44 pH units (0.29% increase per pH unit), while no significant variation was observed for the *a* axis. Polymorph *R*3_T3R3_ exhibited a 0.12% (∼0.1 Å) increase along the *a* axis and a 0.15% (∼0.06 Å) increase along the *c* axis over 0.60 pH units (0.20% and 0.25% increase per pH unit, respectively). Lastly, polymorph *I*2_1_3 exhibited a 0.25% (∼0.2 Å) increase along the *a* axis over 0.80 pH units (0.31% increase per pH unit).

The extracted unit-cell parameters for each sample are reported in Supplementary Tables S2 and S3.

### 3.2. Cubic human insulin structure

**Figure 4:**
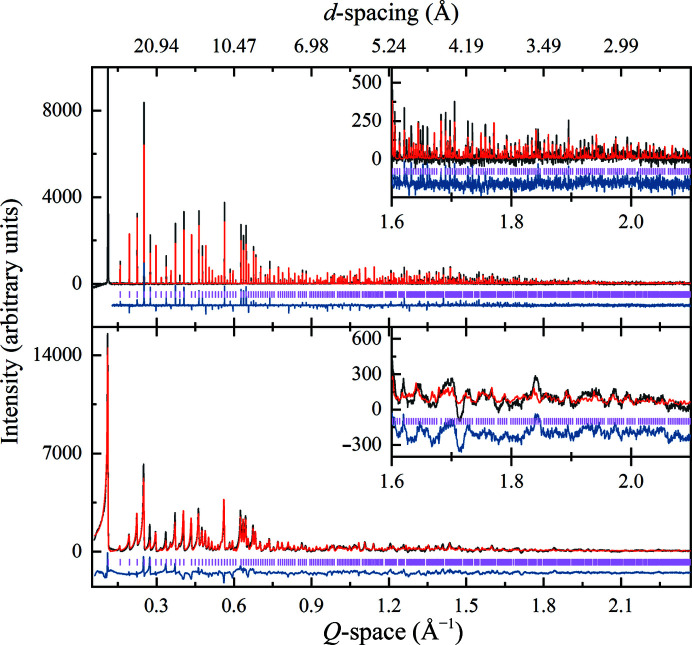
Multi-profile and stereochemically restrained Rietveld refinement of polycrystalline human insulin (polymorph *I*2_1_3). A synchrotron [upper panel; ID22 at ESRF, λ = 1.29974 (1) Å] and a laboratory [lower panel; Malvern Panalytical X’Pert Pro, λ = 1.540585 (3) Å] profile are presented. Upper panel: final pH 8.43, *R*_p_ = 7.70%, *R*_wp_ = 10.98%. The first peak was excluded from the refinement due to artificial distortions caused to the structure. Lower panel: final pH 7.84, *R*_p_ = 2.12%, *R*_wp_ = 3.34%. Black, red and blue lines represent the experimental data, the calculated profile and the difference between them, respectively. The vertical lines correspond to indexed peak positions. Data collection was performed at room temperature. A magnification of the high *d*-spacing resolution region of each data set is shown as an inset.

**Figure 5:**
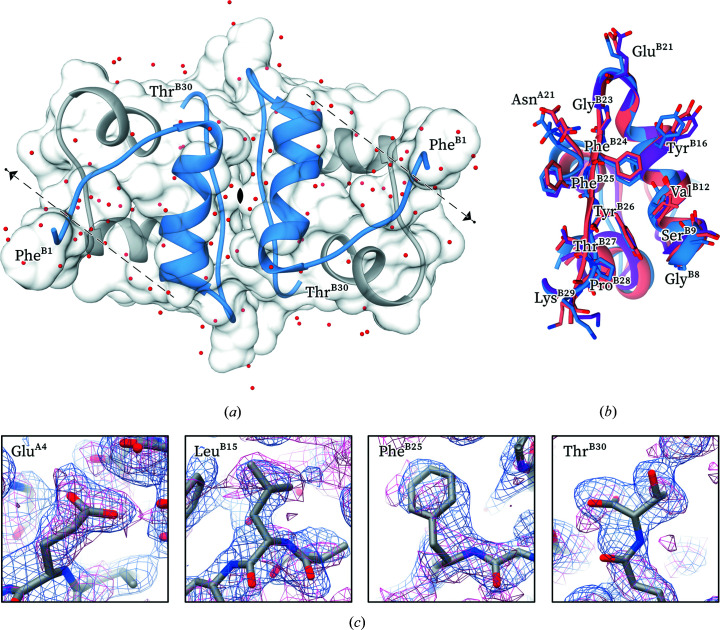
(*a*) The polycrystalline T_2_ insulin dimer structure (PDB entry 7qac). The relevant symmetry elements are shown. Type A and type B chains are shown in white and blue, respectively. (*b*) Overview of the residues involved in the dimerization interface. Blue, polycrystalline cubic structure (PDB entry 7qac); purple, single-crystal cubic structure (PDB entry 3i40); pink, rhombohedral T_6_ structure (PDB entry 1mso). (*c*) 2*F*_o_ − *F*_c_ (blue) and OMIT (magenta) maps contoured at 1σ around specific residues of the polycrystalline structure.

The crystal structure of the cubic human insulin polymorph was obtained *via* a multi-profile and stereochemically restrained Rietveld refinement (Fig. 4 ▸) at 2.7 Å resolution (total *R*_wp_ = 6.94% and total χ^2^ = 2.06). The unit-cell parameter *a* was determined to be 78.86015 (7) Å. Overall, 18 158 reflections were extracted from the six XRPD patterns and 1951 restraints were imposed on the structural model. A total of 63 water molecules were identified in 2*F*_o_ − *F*_c_ and OMIT maps and an overall reasonable model fit to the electron density was observed (Fig. 5 ▸ *c*). The final model yielded a *MolProbity* score of 3.78 (Chen *et al.*, 2010 ▸; Williams *et al.*, 2018 ▸). Detailed statistics can be found in Table 2 ▸.

The model obtained revealed the T_2_ dimeric conformation of insulin (Fig. 5 ▸ *a*) in line with earlier studies [PDB entries 9ins (Gursky, Li *et al.*, 1992 ▸), 3i40 and 3i3z (Timofeev *et al.*, 2010 ▸)]. The asymmetric unit contains a single insulin molecule, which forms a dimer with a symmetry-related molecule. The *I*2_1_3 polymorph exhibits the largest observed solvent content [∼65%(*v*/*v*)] of all insulin polymorphs, followed by the hexameric *C*222_1_ polymorph (PDB entry 2om1; Norrman & Schluckebier, 2007 ▸; Karavassili *et al.*, 2012 ▸). Contacts between insulin dimers extend in all three directions along the twofold screw axes, forming solvent channels parallel to each crystallographic axis, but also parallel to the threefold diagonal axis.

The insulin dimers in the *I*2_1_3 unit cell are mostly stabilized *via* an antiparallel β-sheet consisting of the two C-terminal regions of chain B of each monomer. This dimer interface involves residues AsnA21, GlyB8, SerB9, ValB12, TyrB16, GluB21 and GlyB23–LysB29. The hydrogen-bonding network formed between these residues is highly conserved in all cubic insulin structures, as well as in the hexameric assemblies. Despite an overall all-atom r.m.s.d. of 1.84 Å, no significant deviations in backbone or side-chain atoms are observed between the single-crystal (PDB entry 3i40) and the polycrystalline (PDB entry 7qac) cubic HI models for the residues participating in the dimer interface (Fig. 5 ▸ *b*), given the 2.7 Å resolution of the polycrystalline structure.

### 3.3. Rietveld refinement at high symmetries

Typically, in macromolecular powder diffraction multiple data sets are employed for structure refinement to exploit the variation in unit-cell parameters which results in anisotropic peak shifts and allows partial deconvolution of overlapping peaks. Such variation in unit-cell parameters can be a result of thermal variation (Brunelli *et al.*, 2003 ▸; Shankland *et al.*, 1997 ▸), controlled radiation damage (Besnard *et al.*, 2007 ▸; Margiolaki *et al.*, 2013 ▸) or variation in crystallization conditions (for example pH; Basso *et al.*, 2005 ▸, 2010 ▸; Margiolaki *et al.*, 2007 ▸), as was the case in the present study. However, the cubic crystal system exhibits exact peak overlap, and therefore any lattice variation results in an isotropic peak shift without any deconvolution of overlapping peaks. This makes it a remarkably challenging case for structure refinement, especially for such a complex molecule.

In this study, we performed combined synchrotron and laboratory refinement of a protein structure. Due to the lower photon flux of laboratory instruments samples can be irradiated for more than 24 h, resulting in excellent counting statistics even in the highest resolution shells. On the other hand, the unparalleled angular resolution of synchrotron diffractometers is crucial for deconvolution of the heavily overlapping peaks in protein samples, especially in the early stages of indexing and structure solution. In the absence of a single instrument with excellent angular resolution, which mitigates radiation-related sample degradation, this combinatorial approach of synchrotron and laboratory diffraction data for protein structure refinement has proven to be of great value for macromolecular powder diffraction.

Even though a multi-profile Rietveld refinement does not alleviate peak overlap in the case of cubic symmetry, it does however increase the information content available for refinement in terms of *d*-spacing resolution and counting statistics. In contrast to our initial concerns, the refinement process proved to be robust and relatively rapid, overcoming the expected difficulties of powder crystallography in such high symmetries.

## 4. Discussion

### 4.1. pH-driven conformational transitions

A variety of solution studies have demonstrated that insulin hexamers exist in an equilibrium between the T_6_, 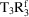 and R_6_ states (Renscheidt *et al.*, 1984 ▸; Wollmer *et al.*, 1987 ▸; Thomas & Wollmer, 1989 ▸; Roy *et al.*, 1989 ▸; Brader *et al.*, 1991 ▸; Gross & Dunn, 1992 ▸; Choi *et al.*, 1993 ▸). Although T_6_ is the predominant state, the equilibrium can be shifted towards the 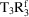 state by the addition of anions and towards the R_6_ state by the addition of phenol derivatives. In this study, we have observed two phase transitions that are entirely pH-driven.

Although the crystallization solution contains thiocyanate ion, a strongly chaotropic anion that is capable of stabilizing the B1–B8 helix in the R state (Whittingham *et al.*, 1995 ▸), the T_6_ conformation of insulin is observed at pH < 6.77, while a further increase in the pH seems to favor the 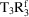 state. More interestingly, at pH > 7.35 zinc-free cubic crystals of dimers are observed. Importantly, in contrast to all other cubic insulin structures available in the literature, in this study the cubic crystals were grown in the presence of zinc.

**Figure 6:**
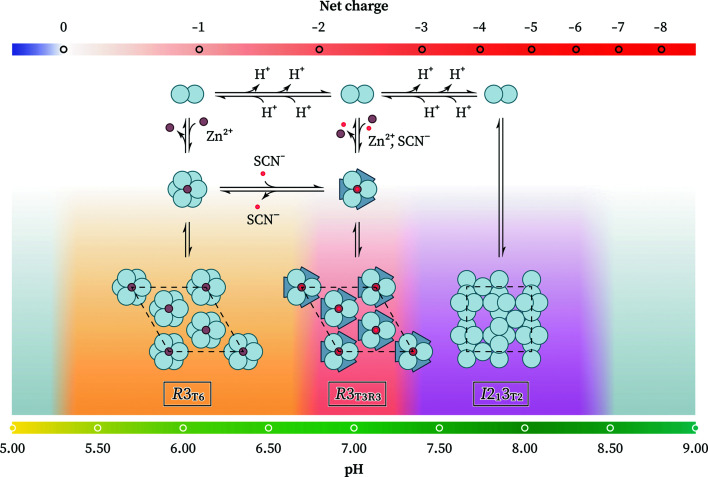
Mechanistic hypothesis behind the pH-driven conformational transitions of insulin.

As the pH increases from 5.5 to 8.5, insulin acquires a progressively more negative overall charge (pI 5.3) and thus increasingly stronger electrostatic repulsions develop between the insulin molecules. The effective charge of insulin is considerably affected by the ionic strength of the solution, as well as the oligomerization state, which alters the microenvironment of certain residues. We hypothesize that at the 2.1 Zn^2+^:insulin hexamer ratio used in this study the overall repulsions between insulin dimers at higher pH overcome the attractive forces of the zinc ions that coordinate with three HisB10 to form hexamers, thus favoring the formation of dimeric cubic crystals. We speculate that an increase in the zinc content would shift the equilibrium towards the hexameric state, and thus that the *R*3_T3R3_ polymorph would be the predominant form even at pH > 7.35, but further experiments are required to test this hypothesis. By a similar argument, an increase in thiocyanate content would be expected to induce the *R*3_T6_ → *R*3_T3R3_ transition at lower pH (Fig. 6 ▸).

A potential pH-sensitive candidate residue driving these phase transitions is GluB13. In the hexameric assembly, six GluB13 residues are closely located to each other at the center of the hexamer. Given a p*K*_a_ of 4.3, the six glutamate residues are likely to be mostly deprotonated and negatively charged at physiological pH; thus, the strongly attractive interaction between zinc ions and three HisB10 residues is necessary for the formation of hexamers. It has previously been shown that the binding of calcium ions in the central hexamer cavity counteracts most of the repulsive GluB13 interactions and further stabilizes the hexameric Zn-insulin assembly (Storm & Dunn, 1985 ▸).

**Figure 7:**
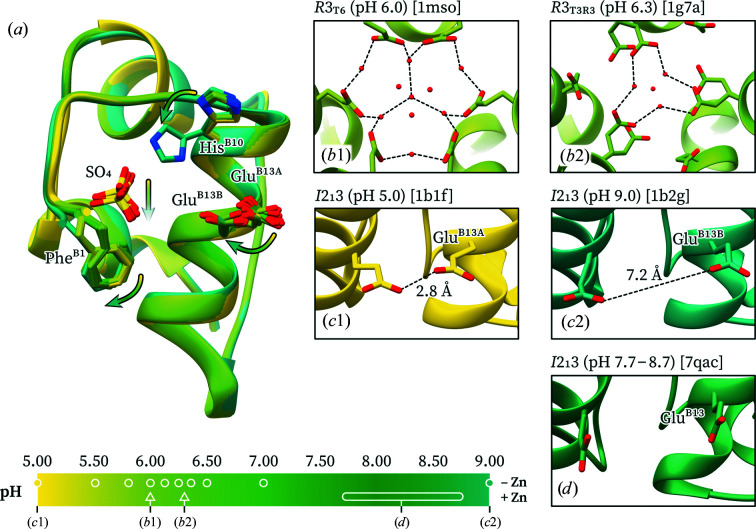
Variation of the GluB13 conformation with pH. (*a*) Overlay of ten cubic insulin structures from pH 5.0 to 9.0. The residues that undergo major structural changes are highlighted. The arrows indicate the trend upon pH increase. (*b*1) Six GluB13 residues in the T_6_ hexamer form a highly ordered hydrogen-bond network with neighboring water molecules. (*b*2) In the 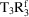 hexamer three of the GluB13 residues exhibit double conformations, while the other three are retracted away from the hexamer core. (*c*1) At pH 5.0, the two GluB13 residues of the cubic polymorph are hydrogen-bonded (proximal conformation). (*c*2) At pH 9.0, the GluB13 residues in the cubic polymorph have completely switched to a new conformation that considerably separates them (distant conformation). (*d*) In the polycrystalline cubic structure the two GluB13 residues are found in the distant conformation. Structures are colored based on pH. PDB codes are shown in parentheses.

At pH 6.0 the six GluB13 residues form a well defined hydrogen-bond network in the T_6_ state (PDB entry 1mso; Smith *et al.*, 2003 ▸), while in the 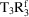 and R_6_ states the GluB13 side chains are less well ordered and are typically found in multiple conformations, suggesting a role contributing to the T → R transition (Fig. 7 ▸ *b*). In fact, mutation of GluB13 to Gln yields 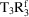 hexamers at pH 7.0 even in the absence of zinc or other helix-stabilizing anions, further supporting this hypothesis (Bentley *et al.*, 1992 ▸).

Progressively increasing electrostatic repulsions between GluB13 with increasing pH have also been observed crystallographically in titration studies of cubic insulin crystals (Diao, 2003 ▸). At pH < 5.8 the two symmetry-related GluB13 residues in the insulin dimer are hydrogen-bonded (proximal conformation, 2.8 Å distance; PDB entry 1b17). At pH 6.0–6.98 a second, distant conformation is observed with a progressively increasing occupancy, while at pH 9.0 only the second conformation is present, in which the two GluB13 side-chain carboxylates are located 7.2 Å apart (distant conformation; PDB entry 1b2g; Fig. 7 ▸ *a*). Neutron diffraction studies on the cubic polymorph have confirmed that GluB13 is deprotonated at pD 9.0 (pH ≃ 8.6; PDB entry 2zpp; Ishikawa, Chatake, Morimoto *et al.*, 2008 ▸). In this study (final pH 7.70–8.70) the two GluB13 residues in the cubic polymorph are found in the distant conformation (Fig. 7 ▸ *d*), which is consistent with the repulsion hypothesis.

Taken together, the previous work discussed above and the data from this study suggest that pH-dependent deprotonations are the driving force of the hexamer-to-dimer oligomerization transition and the related shift in crystal form (from rhombo­hedral to cubic) at pH 7.35.

### 4.2. Conformational plasticity of insulin

Although found in an extended conformation, the N-terminal region of chain B of the cubic polymorph does not adopt the same conformation as in the hexameric T state. While the T conformation is mostly preserved up to HisB5, a sharp double bend is observed at GlnB4 and AsnB3 which tethers PheB1 closer to the insulin core, residing in a hydrophobic cavity comprised of LeuA16, ValB2, AlaB14, LeuB17 and ValB18. In particular, the distance between PheB1 C^ɛ1^ and AlaB14 C^β^ contracts from 11.2 Å in the hexagonal T_6_ state (PDB entry 1mso) to only 4.5 Å in the cubic polymorph (PDB entry 3i40).

In the case of the polycrystalline cubic insulin structure reported here (PDB entry 7qac) only a single bend is observed at AsnB3, resulting in a semi-extended conformation of chain B (Table 3 ▸). Rather than retracting towards the α-helix of chain B, PheB1 is instead anchored to a hydrophobic interface formed by two symmetry-related PheB1 residues along the crystallographic threefold symmetry axis and a symmetry-related GlnB4. This results in a 7 Å translocation of PheB1 C^α^ with respect to the single-crystal cubic structure (PDB entry 3i40). Although the N-terminal fold of chain B is quite conserved among various cubic insulin structures, human or otherwise, this alternate conformation of the polycrystalline structure could be a result of the translocation of the α^II^ helix of chain A, which transposes LeuA16 about 2 Å further back, leading to an increase in the size of the hydrophobic cavity and potentially allowing ValB2 and PheB1 to untether from it (Supplementary Fig. S2).

**Figure 8:**
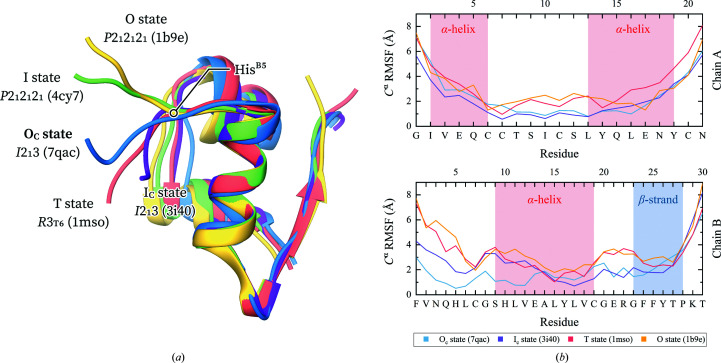
(*a*) Overlay of insulin structures in a variety of T-like states. (*b*) Root-mean-square fluctuations of the C^α^ position during coarse-grained molecular-dynamics simulations. Blue, polycrystalline cubic structure (PDB entry 7qac); purple, single-crystal cubic structure (PDB entry 3i40); pink, rhombohedral T_6_ structure (PDB entry 1mso); green, orthorhombic AlaB8NMeAla T_2_ structure (PDB entry 4cy7); yellow, orthorhombic SerB9Glu T_2_ structure (PDB entry 1b9e).

Apart from the distinct T, R^f^ and R states, the N-terminal region of chain B is capable of folding into a variety of extended, T-like states. Following the previously established nomenclature, the N-terminus can adopt an open, *i.e.* O, state, in which residues B1–B4 are detached from the rest of the molecule (PDB entry 1b9e; Yao *et al.*, 1999 ▸), or an intermediate, *i.e.* I, state, in which residues B1 and B2 are positioned further away from the molecule than in the O state [PDB entries 2ws0 (Jiráček *et al.*, 2010 ▸) and 4cy7 (Kosinová *et al.*, 2014 ▸)]. The fold observed in the cubic structures has previously been referred to as a compact intermediate state, *i.e.* the I_c_ state (Kosinová *et al.*, 2014 ▸). The polycrystalline cubic structure determined here retains the AsnB3 bend of the I_c_ fold; however, since residues B1–B2 extend away rather than towards the hydrophobic AlaB14 core, we describe it as a compact open state (*i.e.* the O_c_ state). In all these T-like states HisB5 acts as a hinge between the highly flexible B1–B4 residues and the rigid B6–B8 residues (Fig. 8 ▸ *a*).

It is therefore evident that although only the T state is observed in solution for the insulin monomer, a wide range of T-like states can be stabilized in a crystal setting (as dimers or hexamers). Our molecular-dynamics simulations on these crystalline structures revealed significant variation in their flexibility. Specifically, the N-terminal region of chain B is highly rigid in the polycrystalline cubic structure compared with the more mobile I_c_ state of other cubic insulin structures. The detached B1–B4 conformation of the T and O states results in higher flexibility of the N-terminus. While the I_c_, O and T states exhibit a mostly invariant mobility after CysB7, the polycrystalline O_c_ state is again more rigid. No significant variation was observed in the flexibility of chain A. Monitoring the LeuB15–PheB24, ValB12–TyrB26 and GlyB8–ProB28 C^α^-atom distances throughout the simulation (Papaioannou *et al.*, 2015 ▸) provided further evidence that the C-terminus of chain B retains the inactive closed conformation (Supplementary Table S4), which is stabilized by hydrophobic interactions with the α-helix of chain B. An open conformation of the C-terminus is required for insulin to bind to its receptor (Lawrence, 2021 ▸).

## 5. Conclusions

In the present study, we explored the polymorphism of human insulin in the presence of zinc and thiocyanate ions in the pH range 4.5–8.5. With increasing pH HI forms T_6_ and 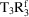 hexamers, while at pH > 7.35 only T_2_ dimers were observed in a cubic crystal symmetry.

The angular and *d*-spacing resolution of the collected XRPD data motivated us to refine the structure of the cubic polymorph by employing a multi-profile and stereochemically restrained Rietveld refinement. The flexible rigid-body description of amino acids in *GSAS* proved to be essential for a robust refinement at 2.7 Å resolution, while we employed synchrotron and laboratory diffraction data in a combined refinement to benefit from the strengths of each instrumentation setup. Despite our concerns due to the high symmetry of the crystals, refinement proved to be feasible.

The polycrystalline cubic insulin structure exhibits some notable differences from other single-crystal structures of the same polymorph, especially in the conformation of the N-terminal region of chain B. We hypothesize that a translocation in LeuA16 increases the size of the hydrophobic cavity which typically houses PheB1, allowing it to adopt a new conformation, termed the O_c_ state. Our molecular-dynamics simulations suggested that this O_c_ state is less flexible than the typical I_c_ state of the cubic polymorph and other T-like states, implying that this is a more rigid conformation of human insulin.

The observed phase transitions are entirely pH-driven. Strikingly, we observed a transition to a zinc-free cubic insulin state despite the presence of zinc in solution. We argue that these transitions are a result of the increase in electrostatic repulsion between neighboring GluB13 residues as a result of pH variation. Further investigations of these pH-driven phase transitions, in terms of varying zinc-to-insulin and thiocyanate-to-insulin ratios, are required to test our hypotheses, as well as single-crystal X-ray diffraction experiments to yield higher resolution structural models.

## 6. Data availability

Atomic coordinates for the polycrystalline cubic human insulin have been deposited in the Protein Data Bank under accession code 7qac. Experimental and calculated diffraction profiles have been deposited in the structure-factors file.

## Supplementary Material

PDB reference: polycrystalline cubic human insulin, 7qac

Supplementary Figures and Tables. DOI: 10.1107/S2059798323001328/tz5109sup1.pdf

## Figures and Tables

**Table 1:** Crystallization parameters

Method	Batch
Container	1.5 ml Eppendorf tubes
Temperature (K)	298
Buffer composition of protein solution	ddH_2_O
Final composition†	13.50 mg ml^−1^ (2.32 m*M*) human insulin, 0.82 m*M* zinc acetate, 10.56 m*M* sodium thiocyanate, 0.4 *M* phosphate (Na_2_HPO_4_/KH_2_PO_4_)
Starting pH	4.50–8.50
Final volume (ml)	1.25

† Values calculated based on the crystallization protocol presented in Section 2.

**Table 2:** Crystallization, data collection, refinement and model-quality statistics of polycrystalline cubic HI (PDB entry 7qac) at 2.7 Å resolution

Sample	nl121	nl218	nl219	nl220	nl221	nl220
Crystallization						
Final batch composition	13.50 mg ml^−1^ insulin, 0.82 m*M* zinc acetate, 10.56 m*M* sodium thiocyanate, 0.4 *M* sodium potassium phosphate (Na_2_HPO_4_/KH_2_PO_4_)					
pH						
Average	8.56	7.88	8.02	8.17	8.26	8.17
Starting/final	8.69/8.43	8.06/7.70	8.30/7.74	8.50/7.84	8.70/7.82	8.50/7.84
Data collection						
Diffraction source	ID22, ESRF					X’Pert Pro
Detector	Nine Si(111) analyzer crystals with LaBr_3_ scintillator detectors					PIXcel 1D
Wavelength (Å)	1.29974 (1)	1.29986 (3)	1.299995 (3)	1.299995 (3)	1.299995 (3)	1.540585 (3)†
Temperature (K)	298	298	298	298	298	298
Total exposure time (min)	2	2	2	2	2	720
Resolution range (Å)	39.43–2.29‡	39.43–2.70‡	39.43–2.70‡	39.43–2.70‡	39.43–2.72‡	55.76–2.64
Refinement						
Space group	*I*2_1_3 (No. 199)					
Unit-cell parameter *a* (Å)	78.86015 (7)					
Lattice strain parameter δ_11_ (Å)	0	0.00031788	0.00087738	−0.00016805	0.00019147	0.0021313
Scale factor	0.18854	0.07749	0.05598	0.12654	0.17352	0.89767
Solvent scattering coefficients						
*A*_S_	6.124	6.121	6.019	6.036	6.052	6.351
*U*_S_	2.739	2.503	2.920	2.859	2.775	6.437
No. of reflections	3816	2345	2349	2345	2305	4998
No. of data points	8256	7003	7002	7002	7003	4740
No. of restraints	1951					
No. of parameters	786					
*R*_p_ (%)	7.70	11.54	12.16	8.80	7.61	2.12
*R*_wp_ (%)	10.98	14.75	15.17	12.12	10.95	3.34
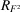 (%)	42.02	38.68	44.09	38.89	35.52	24.00
No. of non-H atoms						
Protein	406					
Solvent	63					
Heterogen	0					
Model quality						
Ramachandran analysis						
Favored (%)	91.49					
Allowed (%)	8.51					
Outliers (%)	0.00					
Rotamer outliers (%)	13.04					
Root-mean-square deviations						
Bond lengths (Å)	0.015					
Bond angles (°)	1.43					
Clashscore	92.88					
*MolProbity* score	3.78					

† Cu *K*α radiation.

‡ Peak (110) was excluded from refinement due to artificial distortions caused to the structure.

**Table 3:** Secondary-structure annotation of several insulin structures with a T-like fold on chain B Annotation was performed using *DSSP* (Touw *et al.*, 2015 ▸; Kabsch & Sander, 1983 ▸).

PDB code	State	Space group	Secondary structure†	Reference
Chain B sequence			FVNQHLCGSH LVEALYLVCG ERGFFYTPKT	
1mso	T	*R*3	...B...THH HHHHHHHHHG GG.EEE....	Smith *et al.* (2003 ▸)
3i40	I_c_	*I*2_1_3	..SS...THH HHHHHHHHHG GG.B...S..	Timofeev *et al.* (2010 ▸)
7qac	O_c_	*I*2_1_3	..S....TTH HHHHHHHHHT TS.....S..	This study
2ws0	I	*I*4_1_22	.......HHH HHHHHHHHHG GG..TT.	Jiráček *et al.* (2010 ▸)
4cy7	I	*P*2_1_2_1_2_1_	.......HHH HHHHHHHHHG GG.B....	Kosinová *et al.* (2014 ▸)
1b9e	O	*P*2_1_2_1_2_1_	.......HHH HHHHHHHHHG GG.EEE....	Yao *et al.* (1999 ▸)

† H, α-helix; G, 3_10_-helix; E, β-sheet; B, isolated β-bridge; T, hydrogen-bonded turn; S, bend.
