# Supplementary material for: The T2 structure of polycrystalline cubic human insulin
Source: Acta Crystallogr D Struct Biol. 2023 Apr 11;79(Pt 5):374–86. doi: 10.1107/S2059798323001328 (PMC10167666; doi:10.1107/S2059798323001328)
Supplement: Supplementary file 1 [file d-79-00374-sup1.pdf]

STRUCTURAL BIOLOGY

Volume 79 (2023)

Supporting information for article:

The T_2 structure of polycrystalline cubic human insulin

Dimitris P. Triandafillidis, Fotini Karavassili, Maria Spiliopoulou, Alexandros Valmas, Maria Athanasiadou, George Nikolaras, Stavroula Fili, Paraskevi Kontou, Matthew W. Bowler, Christos T. Chasapis, Robert B. Von Dreele, Andrew N. Fitch and Irene Margiolaki

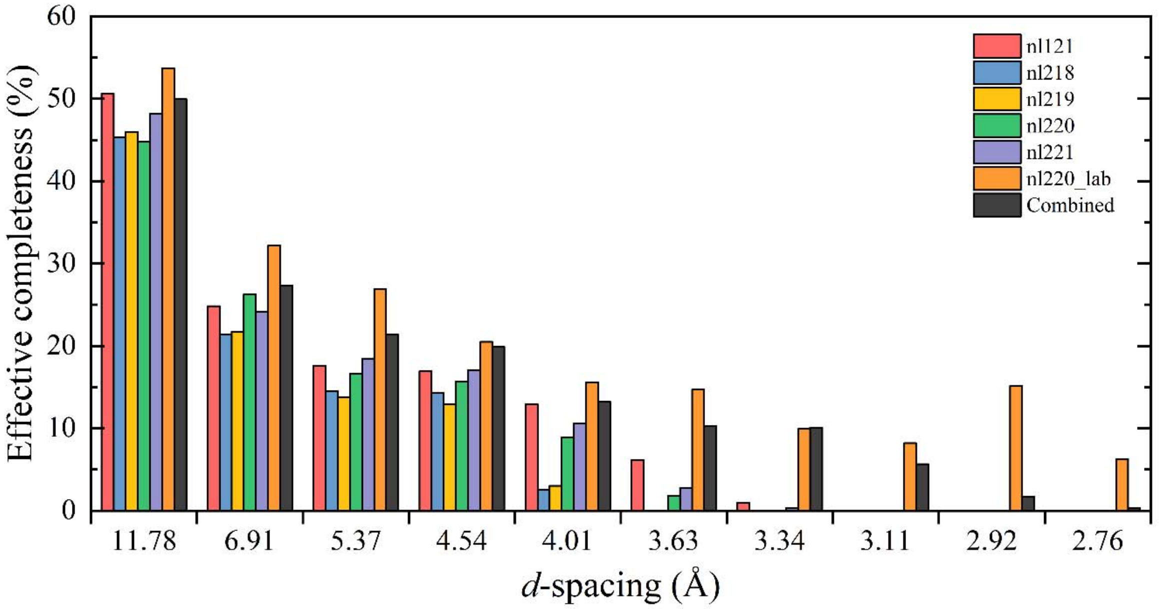

Figure S1 Effective completeness (*i.e.* percentage of reflections with $I/\sigma(I) > 3$) per resolution shell of the single and multi-profile Pawley refinements (non-structure refinement).

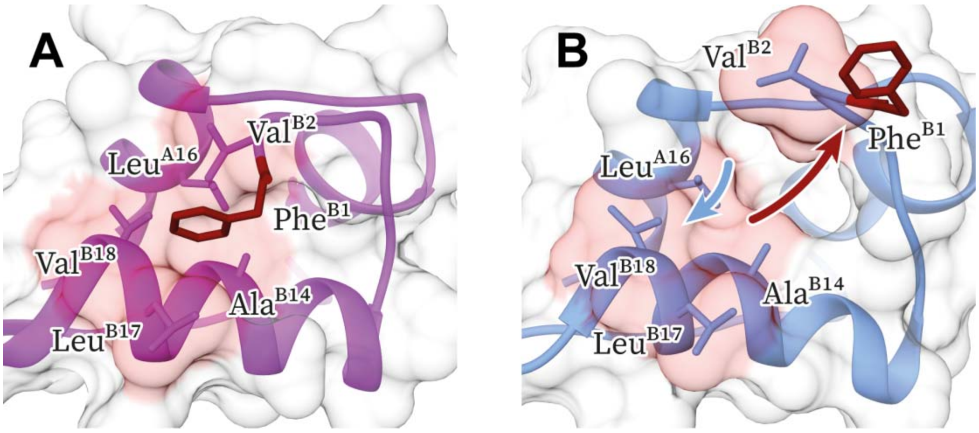

Figure S2 Hydrophobic cavity housing PheB1 in cubic insulin structures. **A:** single-crystal cubic insulin [PDB ID: 3i40 (Timofeev *et al.*, 2010)], **B:** polycrystalline cubic insulin [PDB ID: 7qac].

Table S1 Statistics of single- and multi-profile Pawley refinement using *PRODD* to evaluate the isomorphism of datasets, prior to structure solution and refinement

Statistics of multi-profile Rietveld refinement using *GSAS* are also shown for comparison.

**Single-profile Pawley refinement (*PRODD*)**						
**Dataset**	**nl121**	**nl218**	**nl219**	**nl220**	**nl221**	
R_{wp} (%)	8.267	11.581	12.978	9.369	8.249	
R_{exp} (%)	5.4287	10.6304	11.5334	7.6802	6.2872	
χ^2	2.3192	1.1868	1.2661	1.4881	1.7213	
**Multi-profile Pawley refinement (*PRODD*)**						
**Dataset**	**nl121**	**nl218**	**nl219**	**nl220**	**nl221**	**Total**
R_{wp} (%)	9.757	15.672	18.922	14.235	11.816	12.966
R_{exp} (%)	5.3662	10.5326	11.4273	7.6096	6.2293	7.4824
χ^2	3.3060	2.2141	2.7418	3.4993	3.5980	3.0030
**Multi-profile Rietveld refinement (*GSAS*)**						
**Dataset**	**nl121**	**nl218**	**nl219**	**nl220**	**nl221**	**Total**
R_{wp} (%)	10.98	14.75	15.17	12.12	10.95	6.94
χ^2	- †	- †	- †	- †	- †	2.06‡

† *GSAS* does not calculate χ^2 for each dataset. ‡ Total χ^2 for a stereochemically restrained Rietveld refinement depends not only on fit quality, but also on deviations of stereochemical restraints and their respective weight factors.

Table S2 Lattice parameters and pH values for the first crystallization series of human insulin, as extracted *via* Pawley refinement using *HighScore Plus*.

Data were collected on beamline ID22 (ESRF) [λ = 1.29974(1) Å] at room temperature.

Sample	pH			Space group	Unit-cell parameters						χ^2
	Starting	Final	Average		*a* (Å)	*b* (Å)	*c* (Å)	*α* (°)	*β* (°)	*γ* (°)	
nl11	4.49	5.27	4.88	*amorphous*							
nl12	4.69	5.33	5.01	$R3_{T6}$	82.975(2)	82.975(2)	34.0244(7)	90	90	120	1.3064
nl13	4.89	5.52	5.21	$R3_{T6}$	82.954(2)	82.954(2)	34.0358(7)	90	90	120	1.156
nl14	5.09	5.71	5.40	$R3_{T6}$	82.876(1)	82.876(1)	34.0686(5)	90	90	120	1.2675
nl15	5.31	5.87	5.59	$R3_{T6}$	82.9870(5)	82.9870(5)	34.0604(3)	90	90	120	1.4844
nl16	5.56	6.07	5.82	$R3_{T6}$	82.9869(4)	82.9869(4)	34.0734(2)	90	90	120	1.5197
nl17	5.69	6.14	5.92	$R3_{T6}$	82.9677(4)	82.9677(4)	34.0824(2)	90	90	120	1.6323
nl18	5.92	6.31	6.12	$R3_{T6}$	83.0010(5)	83.0010(5)	34.1082(2)	90	90	120	1.3772
nl19	6.11	6.46	6.29	$R3_{T6}$	83.0031(7)	83.0031(7)	34.1401(4)	90	90	120	1.366
nl110	6.27	6.52	6.40	$R3_{T6}$	82.9949(6)	82.9949(6)	34.1467(3)	90	90	120	1.4791
nl111	6.53	6.76	6.65	$R3_{T6}$	82.9320(8)	82.9320(8)	34.1600(3)	90	90	120	2.351
				$R3_{T3R3}$	80.600(1)	80.600(1)	37.7022(8)	90	90	120	
nl112	6.72	6.82	6.77	$R3_{T6}$	82.929(1)	82.929(1)	34.1628(4)	90	90	120	2.4265
				$R3_{T3R3}$	80.598(1)	80.598(1)	37.6986(6)	90	90	120	
nl113	6.91	6.77	6.84	$R3_{T6}$	82.974(1)	82.974(1)	34.1651(5)	90	90	120	2.099
				$R3_{T3R3}$	80.582(2)	80.582(2)	37.6943(9)	90	90	120	
nl114	7.09	6.84	6.97	$R3_{T3R3}$	80.5817(6)	80.5817(6)	37.6980(3)	90	90	120	1.4038
nl115	7.30	6.93	7.12	$R3_{T3R3}$	80.6179(6)	80.6179(6)	37.7079(3)	90	90	120	1.4232
nl116	7.51	7.06	7.29	$R3_{T3R3}$	80.6304(6)	80.6304(6)	37.7180(3)	90	90	120	1.3602
nl117	7.71	7.11	7.41	$R3_{T3R3}$	80.6461(4)	80.6461(4)	37.7373(2)	90	90	120	1.501
nl118	7.91	7.21	7.56	$R3_{T3R3}$	80.6600(5)	80.6600(5)	37.7443(3)	90	90	120	1.3588
nl119	8.11	7.36	7.74	$R3_{T3R3}$	80.694(3)	80.694(3)	37.759(1)	90	90	120	1.2813
nl120	8.30	7.35	7.87	$I2_13$	78.80(3)	78.80(3)	78.80(3)	90	90	90	1.1682
nl121	8.69	8.43	8.56	$I2_13$	78.9(3)	78.9(3)	78.9(3)	90	90	90	1.5327

Table S3 Lattice parameters and pH values for the second crystallization series of human insulin, as extracted *via* Pawley refinement using *HighScore Plus*.

Data were collected on beamline ID22 (ESRF) [λ = 1.299995(3) Å] at room temperature.

**Sample**	**pH**			**Space group**	**Unit-cell parameters**						χ^2
	**Starting**	**Final**	**Average**		***a*** **(Å)**	***b*** **(Å)**	***c*** **(Å)**	***α*** **(°)**	***β*** **(°)**	***γ*** **(°)**	
nl21	4.42	5.46	4.94	*amorphous*							
nl22	4.64	5.51	5.08	*amorphous*							
nl23	4.80	5.52	5.16	$R3_{T6}$	82.876(1)	82.876(1)	33.9976(4)	90	90	120	1.1800
nl24	5.00	5.63	5.32	$R3_{T6}$	82.674(3)	82.674(3)	34.041(1)	90	90	120	1.4300
nl25	5.20	5.88	5.54	$R3_{T6}$	82.910(1)	82.910(1)	34.0120(6)	90	90	120	1.2867
nl26	5.43	6.11	5.77	$R3_{T6}$	82.831(2)	82.831(2)	34.0660(9)	90	90	120	1.1045
nl27	5.70	6.25	5.98	$R3_{T6}$	82.892(2)	82.892(2)	34.0502(9)	90	90	120	1.1157
nl28	5.90	6.42	6.16	$R3_{T6}$	82.9160(7)	82.9160(7)	34.0599(3)	90	90	120	1.4035
nl29	6.10	6.54	6.32	$R3_{T6}$	82.8705(7)	82.8705(7)	34.1020(3)	90	90	120	1.3628
nl210	6.31	6.65	6.48	$R3_{T6}$	82.898(2)	82.898(2)	34.102(1)	90	90	120	1.2311
nl211	6.55	6.84	6.70	$R3_{T6}$	82.886(2)	82.886(2)	34.1272(9)	90	90	120	1.1997
nl212	6.78	6.98	6.88	$R3_{T3R3}$	80.580(6)	80.580(6)	37.577(2)	90	90	120	1.1757
nl213	7.03	7.15	7.09	$R3_{T3R3}$	80.591(4)	80.591(4)	37.612(3)	90	90	120	1.1898
nl214	7.20	7.28	7.24	$R3_{T3R3}$	80.655(1)	80.655(1)	37.662(1)	90	90	120	1.1919
nl215	7.45	7.47	7.46	$I2_13$	78.7(2)	78.7(2)	78.7(2)	90	90	90	1.0914
nl216	7.70	7.55	7.63	$I2_13$	78.8(3)	78.8(3)	78.8(3)	90	90	90	1.7176
nl217	7.86	7.58	7.72	$I2_13$	78.8390(6)	78.8390(6)	78.8390(6)	90	90	90	1.4283
nl218	8.06	7.70	7.88	$I2_13$	78.8635(3)	78.8635(3)	78.8635(3)	90	90	90	1.6777
nl219	8.30	7.74	8.02	$I2_13$	78.8767(3)	78.8767(3)	78.8767(3)	90	90	90	1.7093
nl220	8.50	7.84	8.17	$I2_13$	78.8344(2)	78.8344(2)	78.8344(2)	90	90	90	2.0488
nl221	8.70	7.82	8.26	$I2_13$	78.9(9)	78.9(9)	78.9(9)	90	90	90	1.4176

Table S4 Distances in residues of the hydrophobic core in the C-terminus of chain B and their mean distance in molecular dynamics simulations.

	**Distance in X-ray structure (Å)**				**Mean distance in simulations (Å)**			
**PDB ID**	**7qac**	**3i40**	**1mso**	**1b9e**	**7qac**	**3i40**	**1mso**	**1b9e**
LeuB15 – PheB24	6.8	6.7	6.8	7.1	7.0(1.0)	6.6(1.4)	6.3(1.8)	6.7(2.0)
ValB12 – TyrB26	6.3	7.1	6.9	7.4	6.5(1.9)	7.4(2.0)	6.7(2.3)	6.9(2.5)
GlyB8 – Pro28	9.8	8.8	9.6	7.4	9.5(2.5)	8.1(2.6)	9.3(2.9)	7.5(3.3)
